# Association of the National Health Guidance Intervention for Obesity and Cardiovascular Risks With Health Outcomes Among Japanese Men

**DOI:** 10.1001/jamainternmed.2020.4334

**Published:** 2020-10-05

**Authors:** Shingo Fukuma, Toshiaki Iizuka, Tatsuyoshi Ikenoue, Yusuke Tsugawa

**Affiliations:** 1 Human Health Sciences, Kyoto University Graduate School of Medicine, Kyoto, Japan; 2 Graduate School of Economics, the University of Tokyo, Tokyo, Japan; 3 Division of General Internal Medicine and Health Services Research, David Geffen School of Medicine at UCLA, Los Angeles, California; 4 Department of Health Policy and Management, UCLA Fielding School of Public Health, Los Angeles, California

## Abstract

**Importance:**

Obesity and cardiovascular risks have become major public health problems. However, evidence is limited as to whether population-level lifestyle interventions for obesity and cardiovascular risk factors are associated with improved population health outcomes.

**Objective:**

To investigate the association of the national health guidance intervention in Japan with population health outcomes.

**Design, Setting, and Participants:**

This cohort study used a regression discontinuity design that included men aged 40 to 74 years who participated in the national health screening program in Japan from April 2013 to March 2018.

**Exposures:**

Assignment to the national health guidance intervention (counseling on healthy lifestyle and appropriate clinical follow-up for individuals found to have waist circumference of 85 cm or greater with 1 or more cardiovascular risk factors during annual national health screening program).

**Main Outcomes and Measures:**

Changes in obesity status (body weight, body mass index, waist circumference), and cardiovascular risk factors (blood pressure, hemoglobin A_1c_ level, and low-density lipoprotein cholesterol level) 1 to 4 years after screening.

**Results:**

Of 74 693 men (mean [SD] age, 52.1 [7.8] years; mean [SD] baseline waist circumference, 86.3 [9.0] cm), the assignment to the health guidance intervention was associated with lower weight (adjusted difference, −0.29 kg; 95% CI, −0.50 to −0.08; *P* = .005), body mass index (−0.10; 95% CI, −0.17 to −0.03; *P* = .008), and waist circumference (−0.34 cm; 95% CI, −0.59 to −0.04; *P* = .02) 1 year after screening. The observed association of the guidance assignment attenuated over time and was no longer significant by years 3 to 4. No evidence was found that the health guidance intervention was associated with changes in participants’ systolic blood pressure, diastolic blood pressure, hemoglobin A_1c_ level, or low-density lipoprotein cholesterol level in years 1 to 4.

**Conclusions and Relevance:**

Among working-age men in Japan, the national health guidance intervention was not associated with clinically meaningful weight loss or other cardiovascular risk factor reduction. Further research is warranted to understand the specific design of lifestyle interventions that are effective in improving obesity and cardiovascular risk factors.

## Key Points

**Question:**

Is the Japanese national health guidance intervention for obesity and cardiovascular risks associated with improved population health outcomes?

**Findings:**

In this national cohort study of 74 693 working-age men in Japan, assignment to the health guidance intervention was associated with a small decrease in weight (−0.29 kg; 95% CI, −0.50 to −0.08) 1 year after the screening, an association that attenuated over time and was no longer significant by years 3 to 4. No evidence was found that the health guidance intervention was associated with changes in blood pressure, hemoglobin A_1c_ level, or low-density lipoprotein cholesterol level in years 1 to 4.

**Meaning:**

Among working-age men in Japan, the national health guidance intervention was not associated with clinically meaningful weight loss or other cardiovascular risk factor reduction; further research is warranted to understand the specific design of lifestyle intervention programs that are more effective in improving population health.

## Teaser

This cohort study investigates the association of the national health guidance intervention for obesity and cardiovascular risks with population health outcomes among working-age men in Japan.

## Introduction

Obesity is an important modifiable risk factor that leads to many diseases, including diabetes, hypertension, dyslipidemia, coronary heart disease, and stroke.^1,2,3^ Globally, an estimated 1.9 billion adults are overweight, and an additional 650 million are obese.^4^ Obesity can potentially be mitigated by lifestyle modifications, including a healthy diet and increased physical activity. Moreover, obesity-related health complications can be substantially reduced by using effective, inexpensive medications. Nevertheless, as many as half of the obese individuals are unaware of the health risks thus incurred.^5^ Consequently, many of them have undiagnosed diabetes and hypertension,^6,7^ indicating that there are missed opportunities to decrease the global burden of disease related to obesity. With the aim of reducing the risk of cardiovascular diseases, screening programs for obesity and cardiovascular risk factors and associated lifestyle intervention programs have been implemented in many countries. However, evidence is limited as to whether population-level screening programs and accompanied lifestyle interventions for obesity and cardiovascular risk factors reduce mortality or the incidence of cardiovascular diseases.^8^

In 2008, Japan introduced a nationwide screening program to identify individuals with high obesity and cardiovascular risks (known as metabolic syndrome) and to provide health guidance to reduce weight and improve cardiovascular risk.^9,10^ All adults aged 40 to 74 years were required by law to participate every year, and approximately 29 million people in Japan received the screening program in 2017.^11^ An important feature of the national program is that, in addition to screening individuals, it provides lifestyle intervention programs for patients at high cardiovascular risk, which is more intensive than many similar programs in other countries. Given that many other countries, employers, and insurers globally are considering similar lifestyle intervention programs to improve population health and lower health expenditures,^12,13^ it is important to study the impact of this national health guidance intervention using a robust, quasi-experimental design.

We investigated the association of the assignment to the health guidance intervention on participants’ health outcomes among working-age men who participated in the Japanese national screening program. To estimate the association of the health guidance intervention with health outcomes, we used a quasiexperimental regression discontinuity (RD) design. This approach takes advantage of the fact that participants who fall just above or below an arbitrary set threshold value of a continuous variable (waist circumference) are similar in every aspect except for that only those whose waist circumference was above the threshold had a higher probability of assignment to the intervention (the national health guidance intervention).

## Methods

### Data Source

We analyzed a nationwide cohort with annual health screening data between April 2013 and March 2018 from one of the largest employment-based health insurers in Japan (the national sample of employees of civil engineering and construction companies). The database includes information on demographic characteristics (age and sex), obesity status (weight, body mass index [BMI], and waist circumference), cardiovascular risk factors (systolic and diastolic blood pressure, hemoglobin A_1c_ [HbA_1c_] level, and low-density lipoprotein [LDL] cholesterol level), medication use, and lifestyle (smoking status, alcohol use, and exercise habits). Baseline variables were measured using the results of the first health screening in 2014. Health outcomes were measured during the health screening in subsequent years (2015-2018). This study followed the Strengthening the Reporting of Observational Studies in Epidemiology (STROBE) reporting guideline.

### National Health Guidance Intervention in Japan

The screening program consists of multiple steps to identify high-risk populations and provide counseling for adopting healthy lifestyles and seeking medical treatment (ie, the health guidance intervention) to those participants identified as being at high risk. Participants with waist circumferences greater than the sex-specific thresholds (85 cm for men and 90 cm for women) and had 1 or more cardiovascular risk factors (hypertension, diabetes, or dyslipidemia) were required to undergo the health guidance intervention (in addition to receiving a summary report of screening results). Those who were taking antihypertensive, antidiabetic, and antihyperlipidemic drugs—individuals who presumably are cared for and given guidance by clinicians—were not required to undergo the health guidance intervention. Participants who did not meet these criteria received a summary report of screening results via mail (did not undergo the health guidance intervention). The insurers used mail or telephone calls to reach out to participants who were assigned to the health guidance intervention (ie, those determined to be at high risk).

Japan’s national health guidance intervention includes content related to exercise, diet, and medical visits. The intervention is provided by trained instructors supervised by physicians, public health nurses, and dietitians (many instructors themselves are qualified as dietitians or public health nurses). The health guidance intervention was provided through an initial interview by the instructor (individual support ≥20 minutes or group support ≥80 minutes), followed by continuous support for a duration of 3 months or more if determined necessary by the assigned instructor based on the participant’s cardiovascular risk factors (eAppendix A in the Supplement). For those participants who still have a waist circumference greater than the threshold (plus 1 or more risk factors) after receiving the health guidance intervention in the prior year, another health guidance intervention would be provided as a de novo intervention (not as a continuation of guidance provided in the first year). The government subsidizes the cost of the guidance conducted by insurers. The estimated cost of the health guidance intervention was $150 million (1 US dollar = 106 Japanese yen) per year.^14^ More details about the government’s guideline for the national health guidance intervention is available in eAppendix A and eFigure 1 in the Supplement.

### Participants

Among 127 322 men aged 40 to 74 years who were eligible for the screening program, 102 764 (80.7%) received baseline screening. We excluded participants without follow-up screening (n = 11 684). After excluding those with any missing covariates (n = 16 387), we analyzed 74 693 men (eFigure 2 in the Supplement). We focused on the working-age male population because of the small number of women who were corporate employees (n = 11 235), of which only a small proportion (11%) met criteria to receive the health guidance intervention. Nevertheless, as a secondary analysis, we also examined the association of the health guidance intervention with health outcomes among female employees.

### Health Outcomes

Our main outcomes were changes in obesity status—body weight, BMI, and waist circumference—1 year after the screening program. Our secondary outcomes were changes in cardiovascular risk factors 1 year after the screening program—systolic blood pressure, diastolic blood pressure, HbA_1c_ level, and LDL cholesterol level. We also examined longer term (2-4 years after the screening) association of the national health guidance intervention (using the 2016-2018 data).

### Statistical Analysis

To estimate the association of the health guidance intervention with health outcomes, we used a quasiexperimental RD design. The RD design takes advantage of clinical or policy decision rules in which participants are differentially assigned to interventions or control groups if they fall above or below an arbitrary cutoff for a continuous variable.^15,16,17,18,19^ In this study, we used the RD model with waist circumference as the assignment variable, noting that participants with waist circumferences above the arbitrary cutoff (85 cm) had a higher probability of receiving an intervention (ie, health guidance intervention) relative to those with waist circumferences below this cutoff. The RD design compares individuals whose value of the assignment variable (waist circumference) is within the selected bandwidth (6 cm in our study) just above vs below the cutoff level. The RD method is appropriate in this case because individuals who fell just above or below the cutoff value were similar in most aspects except whether they received the intervention. The RD design is preferable to a difference-in-differences method because the latter has an untestable assumption that the outcome variable of treatment and control groups follow parallel trajectories in the absence of the intervention. In sharp RD designs, the value of the assignment variable deterministically determines whether participants receive the intervention; the receipt of intervention is probabilistically determined in fuzzy RD designs.^20^ In this study, we used the fuzzy RD design because the assignment to health guidance intervention was determined based not only on the value of waist circumference, but on several other factors (eAppendix C in the Supplement). Our data confirmed that the probability of assignment to the health guidance intervention changed dramatically at the threshold level of waist circumference, supporting the validity of our method (eFigure 3 in the Supplement).

In our main RD model, we used a local linear RD estimation with robust bias-corrected CIs to avoid overfitting of the data.^21^ To account for potential differences in other characteristics around the threshold of waist circumference, we adjusted for participants’ age, current smoking status (yes/no), alcohol use (not every day; every day, small amount; or every day, large amount), exercise habit (yes/no), systolic and diastolic blood pressure, HbA_1c_ level, LDL cholesterol level, and medication use (indicator variables for antihypertensive drugs, antidiabetic drugs, and antihyperlipidemic drugs) at baseline (measured during the initial screening). We implemented the bias-corrected nonparametric inference procedure, which would be robust to wide bandwidth selection.^22^ In the RD model, we used a triangular kernel function, which gave more weight to participants near the threshold level.

The primary focus of this study was to examine the association of the assignment to the health guidance intervention with health outcomes (ie, the intention-to-treat effect). However, we were also interested in the association of the actual receipt of the health guidance intervention with outcomes (ie, the treatment-on-the-treated [ToT] effect). Data on actual receipt of the health guidance intervention were available only for 2017 to 2018; therefore, we investigated the association of the receipt (the ToT effect) of the health guidance intervention in 2017 with health outcomes in 2018 using the RD model.

### Secondary Analyses

We conducted several secondary analyses. First, we investigated how the probability of assignment to the health guidance intervention changed around the cutoff value of the participants’ waist circumferences. Second, we tested whether the density of waist circumference changed smoothly at the threshold using the McCray test.^23^ Third, to test the smooth continuity of observed covariates at the threshold level of waist circumference, we conducted the RD model using covariates as the outcome variable and waist circumference as the explanatory variable. Fourth, we varied bandwidth to test the robustness of our findings based on the selection of bandwidth. Fifth, to evaluate whether our findings were sensitive to the selection of adjustment variables in the RD model, we reanalyzed the data without adjusting for covariates. Sixth, to investigate the effect of some participants having received the same health guidance intervention in the prior year, we reanalyzed the data, restricting our sample to participants who were not assigned to the health guidance intervention in 2013. Seventh, the data on health outcomes were missing for 11.4% (11 684 of 102 764) of participants due to loss to follow-up. To test how this affects our findings, we conducted a weighted RD analysis in which weights were generated on the basis of the inverse probability of health outcome data being observed.^23^ Eighth, as a falsification test, we conducted the RD assessing the association of assignment to the health guidance intervention in 2014 with their health outcomes in 2013. Ninth, we examined the impact of the health guidance intervention on changes in the proportion of participants taking relevant drugs (antihypertensive, antidiabetic, and antihyperlipidemic drugs), their smoking status, and exercise habits. Finally, to test whether the association of the health guidance intervention with health outcomes varies between men and women employees, we also investigated the association of the health guidance intervention with health outcomes among working women.

All tests were 2-sided; *P* values less than .05 were considered statistically significant. All analyses were performed using Stata, version 16.1 (StataCorp).

### Ethical Review of Study

The institutional review board of Kyoto University approved all study procedures (approval No. R0817). The institutional review board waived informed consent for participants owing to the use of deidentified data.

## Results

### Participant Characteristics

A total of 74 693 men were included in our RD analysis (39 161 within bandwidth). The mean (SD) age was 52.1 (7.8) years; the mean (SD) waist circumference was 86.3 (9.0) cm; and the mean (SD) BMI was 24.5 (3.4) at baseline. Table 1 summarizes the characteristics of participants within the optimal bandwidth of waist circumference. We found no evidence of discontinuity for observed covariates at the threshold of waist circumference, suggesting the smooth distribution of observed covariates at the threshold (eAppendix E and eTable 1 in the Supplement). These results support the validity of the quasirandomization of participants to the intervention and control groups on both sides of the cutoff value. We found that 15.9% of participants (6176 of 38 894) who were assigned to the health guidance intervention actually received the guidance in 2017 (the proportion of the participants who complied with the requirement to receive health guidance intervention).

**Table 1.** Participant Characteristics in the Total Sample and Participants Within Optimal Bandwidths

Characteristic	Mean (SD)		
	Total (n = 74 693)	Waist circumference within bandwidth of 6 cm from the threshold	
		−6 to <0 cm (n = 19 818)	0 to ≤6 cm (n = 19 343)
Age, y	52.1 (7.8)	52.1 (7.8)	52.8 (7.9)
Baseline obesity status			
Waist circumference, cm	86.3 (9.0)	82.2 (1.6)	87.7 (1.7)
Body weight, kg	71.4 (11.0)	66.8 (5.0)	72.45 (5.4)
Body mass index^a^	24.5 (3.4)	23.1 (1.5)	24.8 (1.6)
Baseline cardiovascular risk factors			
Blood pressure, mm Hg			
Systolic	126.5 (16.3)	124.9 (15.9)	127.1 (15.6)
Diastolic	79.6 (11.3)	78.5 (11.1)	80.2 (10.8)
Hemoglobin A_1c_, %	5.7 (0.8)	5.6 (0.6)	5.7 (0.7)
LDL cholesterol, mg/dL	128.1 (31.7)	127.9 (31.2)	130.8 (31.7)
Baseline lifestyle variables, No. (%)			
Current smoking	27 098 (36.3)	6884 (34.7)	6895 (35.6)
Drinking alcohol, No. (%)			
Not every day	40 752 (54.6)	10 300 (52.0)	10 107 (52.3)
Every day, small amount	22 607 (30.3)	6445 (32.5)	6123 (31.7)
Every day, large amount	11 334 (15.2)	3073 (15.5)	3113 (16.1)
Exercise habits	32 259 (43.2)	9059 (45.7)	8324 (43.0)
Baseline medication, No. (%)			
Antihypertensive drugs	14 762 (19.8)	2831 (14.3)	4205 (21.7)
Antidiabetic drugs	4777 (6.4)	845 (4.3)	1185 (6.1)
Antihyperlipidemic drugs	8180 (11.0)	1730 (8.7)	2290 (11.89)

Abbreviation: LDL, low-density lipoprotein.

^a^ Calculated as weight in kilograms divided by height in meters squared.

SI conversion factors: To convert LDL cholesterol to mmol/L, multiply by 0.0259. To convert percentage of total hemoglobin to proportion of total hemoglobin, multiply by 0.01.

### Distribution of Baseline Waist Circumference

Waist circumference was distributed with a median (interquartile range) of 85.5 (80.3-91.5) cm, and 53.1% of participants (54 548 of 102 764) had waist circumferences above the threshold. The smooth distribution (no evidence of manipulation) of waist circumference around the threshold level is shown in eFigure 4 in the Supplement.

### Association of the National Health Guidance Intervention With Health Outcomes

**Figure 1.**
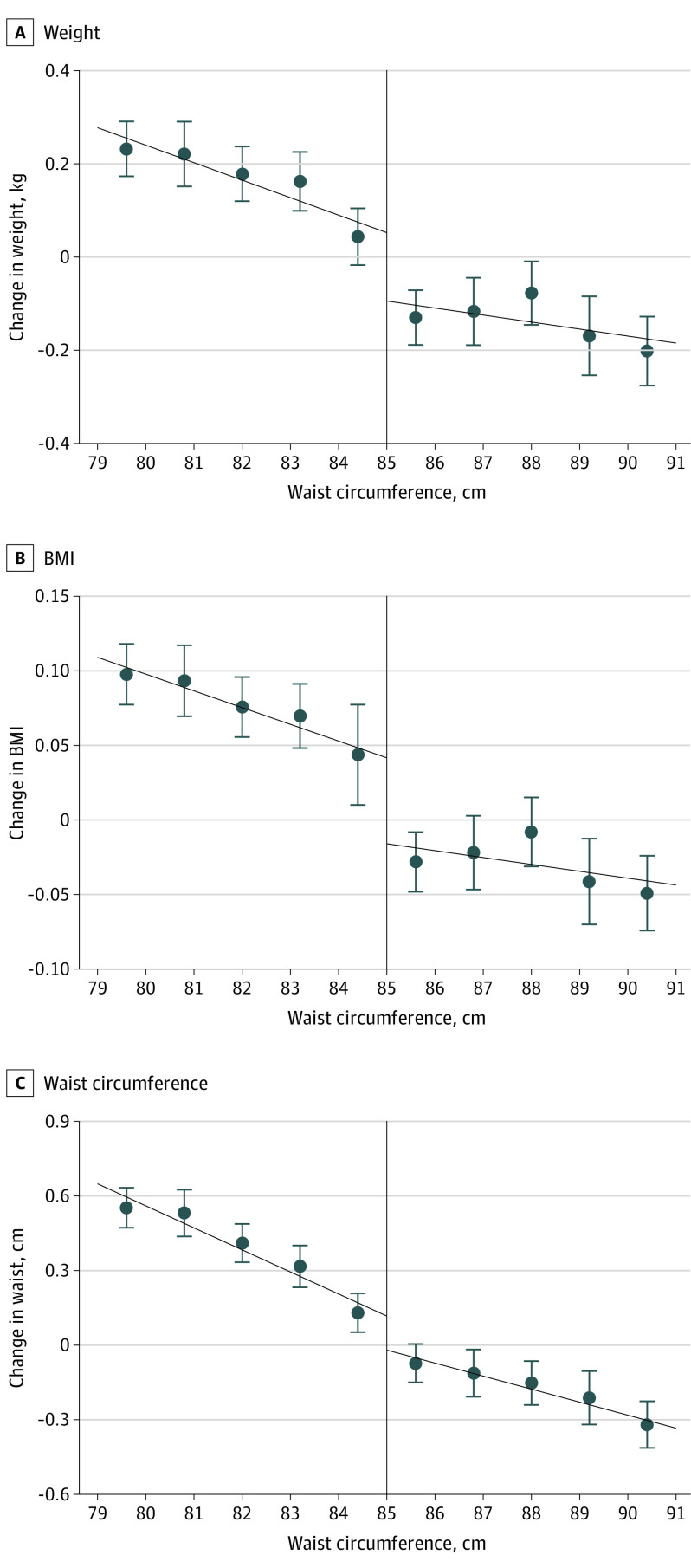
Change in Obesity Status 1 Year After the Initial Screening According to Baseline Waist Circumference Within the Optimal Bandwidths The dots and error bars indicate point estimates and 95% CIs, respectively. The vertical solid line indicates the threshold level of waist circumference. Body mass index (BMI) is calculated as weight in kilograms divided by height in meters squared.

Figure 1 shows the RD plots of change in obesity status (weight, BMI, and waist circumference) around the threshold level. We observed a sharp downward discontinuity in the changes in weight, BMI, and waist circumference. We found that the assignment to health guidance intervention was associated with lower weight (adjusted difference, −0.29 kg; 95% CI, −0.50 to −0.08; *P* = .005), BMI (−0.10; 95% CI, −0.17 to −0.03; *P* = .008) and waist circumference (−0.34 cm; 95% CI, −0.59 to −0.04; *P* = .02) 1 year after screening (Table 2). The observed weight loss attenuated over time, and it was no longer significant by years 3 to 4.

**Table 2.** Association of Assignment to the Health Guidance Intervention With Health Outcomes Using Fuzzy Regression Discontinuity Design^a^

Outcome								
	Main		Long term					
	1 y after screening (2015) (n = 39 161)	*P* value	2 y after screening (2016) (n = 34 293)	*P* value	3 y after screening (2017) (n = 31 400)	*P* value	4 y after screening (2018) (n = 28 975)	*P* value
Change in weight								
Body weight, kg	−0.29 (−0.50 to −0.08)	.005	−0.33 (−0.61 to −0.05)	.02	−0.28 (−0.58 to 0.08)	.13	−0.06 (−0.38 to 0.37)	.96
BMI^b^	−0.10 (−0.17 to −0.03)	.008	−0.10 (−0.20 to −0.01)	.03	−0.10 (−0.20 to 0.02)	.12	−0.01 (−0.12 to 0.14)	.86
Waist circumference, cm	−0.34 (−0.59 to −0.04)	.02	−0.33 (−0.64 to 0.04)	.09	−0.44 (−0.84 to −0.06)	.03	−0.35 (−0.78 to 0.09)	.12
Change in cardiovascular risk factors								
Systolic blood pressure, mm Hg	0.28 (−0.53 to 1.47)	.36	0.26 (−0.97 to 1.66)	.61	−0.36 (−1.83 to 0.90)	.51	−1.16 (−2.76 to 0.17)	.08
Diastolic blood pressure, mm Hg	−0.54 (−1.33 to 0.04)	.07	−0.004 (−0.90 to 0.92)	.98	−0.18 (−1.14 to 0.73)	.67	−0.87 (−2.00 to 0.06)	.06
Hemoglobin A_1c_, %	−0.01 (−0.04 to 0.03)	.74	0.01 (−0.02 to 0.06)	.30	0 (−0.04 to 0.04)	.92	0.02 (−0.02 to 0.07)	.21
LDL cholesterol, mg/dL	0.42 (−1.38 to 2.33)	.62	−0.83 (−3.02 to 1.18)	.39	−0.56 (−3.04 to 1.60)	.54	0.07 (−2.25 to 2.77)	.84

Abbreviations: BMI, body mass index; LDL, low-density lipoprotein.

^a^ We used the bandwidth of regression discontinuity design of 6 cm from the threshold of waist circumference. Analyses were adjusted for age, lifestyle variables (current smoking, alcohol use, exercise habits), systolic blood pressure, diastolic blood pressure, hemoglobin A_1c_ level, LDL cholesterol level, and drug use (antihypertensive drugs, antidiabetic drugs, antihyperlipidemic drugs).

^b^ Calculated as weight in kilograms divided by height in meters squared.

SI conversion factors: To convert LDL cholesterol to mmol/L, multiply by 0.0259. To convert percentage of total hemoglobin to proportion of total hemoglobin, multiply by 0.01.

**Figure 2.**
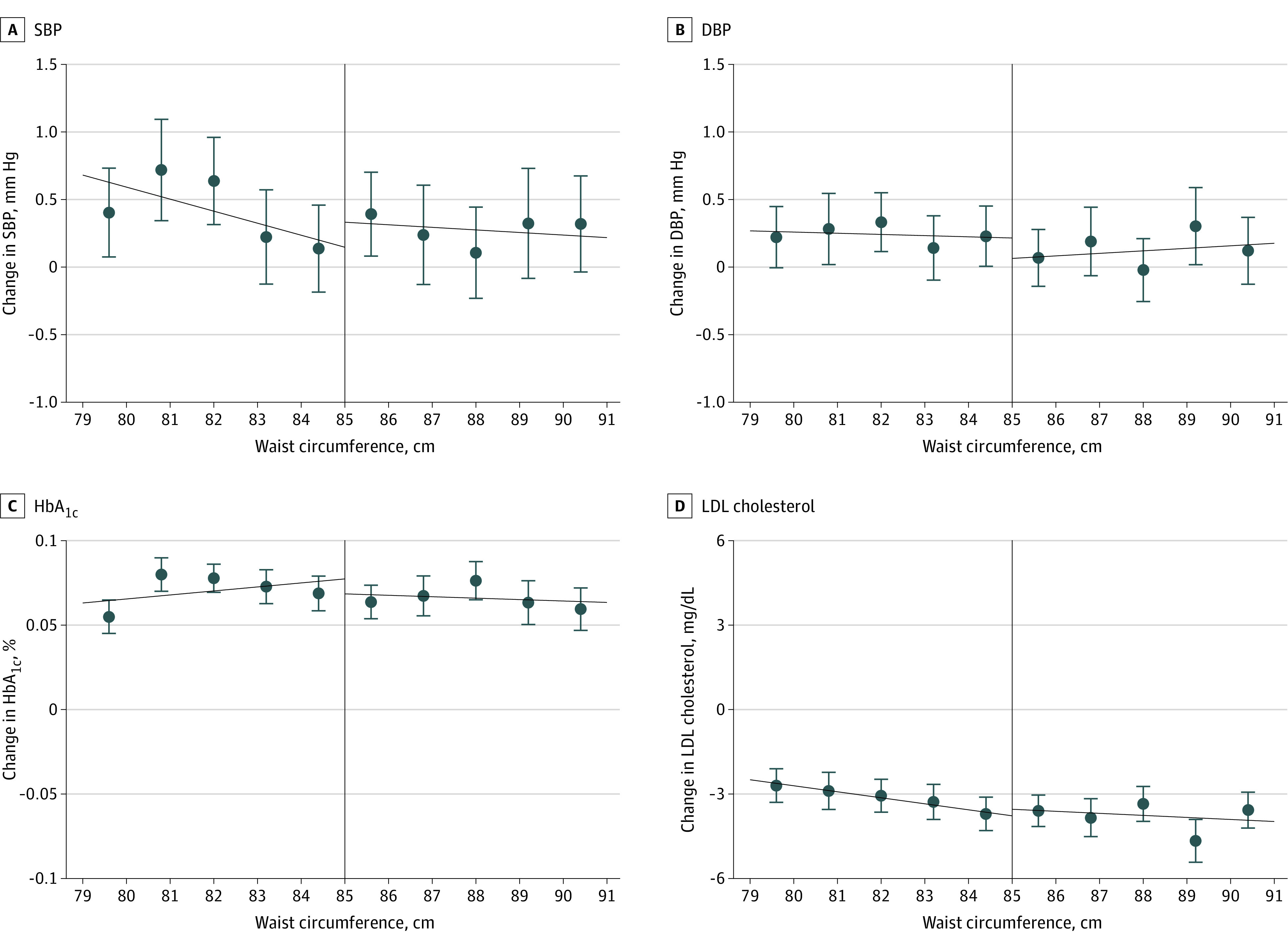
Change in Cardiovascular Risk Factors 1 Year After the Initial Screening According to Baseline Waist Circumference Within the Optimal Bandwidths The dots and error bars indicate point estimates and 95% CIs, respectively. The vertical solid line indicates the threshold level of waist circumference. DBP indicates diastolic blood pressure; HbA_1c_, hemoglobin A_1c_, LDL, low-density lipoprotein; SBP, systolic blood pressure. SI conversion factors: To convert LDL cholesterol to mmol/L, multiply by 0.0259. To convert percentage of total hemoglobin to proportion of total hemoglobin, multiply by 0.01.

Figure 2 illustrates the RD plots of change in cardiovascular risk factors (systolic blood pressure, diastolic blood pressure, HbA_1c_ level, and LDL cholesterol level) around the threshold level. We found no evidence that the assignment to the health guidance intervention was associated with changes in cardiovascular risk factors in 1 to 4 years (Table 2).

The RD analysis of the ToT effect (using participants who did not meet the criteria to receive the guidance as the control group) showed that receipt of the guidance was associated with lower weight (−1.56 kg; 95% CI, −3.10 to −0.22; *P* = .02) and lower BMI (−0.61; 95% CI, −1.19 to −0.14; *P* = .01) 1 year after the screening, whereas we found no evidence that receipt of the guidance was associated with changes in the cardiovascular risk factors (Table 3).

**Table 3.** Association of Actual Receipt of the Health Guidance Intervention With Health Outcomes (2017-2018 Data)^a^

Outcome	1 y after (2018) (n = 39 161)	*P* value
Change in weight		
Body weight, kg	−1.56 (−3.10 to −0.22)	.02
BMI^b^	−0.61 (−1.19 to −0.14)	.01
Waist circumference, cm	−0.44 (−2.03 to 1.69)	.86
Change in cardiovascular risk factors		
Blood pressure, mm Hg		
Systolic	−2.32 (−10.16 to 4.60)	.46
Diastolic	−0.37 (−5.30 to 4.94)	.94
Hemoglobin A_1c_, %	0.10 (−0.10 to 0.31)	.32
LDL cholesterol, mg/dL	6.19 (−4.16 to 20.60)	.19

Abbreviations: BMI, body mass index; LDL, low-density lipoprotein; NA, not applicable.

^a^ We used the bandwidth of regression discontinuity design of 6 cm from the threshold of waist circumference. Analyses were adjusted for age, lifestyle variables (current smoking, alcohol use, exercise habits), systolic blood pressure, diastolic blood pressure, hemoglobin A_1c_ level, LDL cholesterol level, and drug use (antihypertensive drugs, antidiabetic drugs, and antihyperlipidemic drugs).

^b^ Calculated as weight in kilograms divided by height in meters squared.

SI conversion factors: To convert LDL cholesterol to mmol/L, multiply by 0.0259. To convert percentage of total hemoglobin to proportion of total hemoglobin, multiply by 0.01.

### Secondary Analyses

The probability of assignment to the health guidance intervention sharply increased as the participant’s waist circumference rose above the threshold level, as expected (eFigure 3 in the Supplement). The result of the McCray test showed no evidence of manipulation of the waist circumference value by participants or examiners during the screening (eAppendix B in the Supplement). We found no discontinuities in observed covariates at the threshold of waist circumference (eTable 1 in the Supplement).

Our findings were qualitatively unaffected by the use of different bandwidth selections, the analysis without covariates adjustments; restriction of our sample to participants who were not assigned to the health guidance intervention a year before; or accounting for missing data on health outcomes using inverse probability weights in the regression models (eTables 2-5 in the Supplement). The results of our falsification test showed no evidence of the effect of the guidance in 2013 on health outcomes in 2014, as expected (eTable 6 in the Supplement). We found no evidence that health guidance intervention was associated with changes in the rates of drug use, smoking status, and exercise habits (eTable 7 in the Supplement). We found similar results for women, but CIs were larger owing to a smaller sample size (eTable 8 in the Supplement).

## Discussion

Among working-age men who underwent the national health screening program in Japan, we found that the government-implemented health guidance intervention was associated with very small weight loss; the magnitude of weight loss was not clinically meaningful and no longer significant in the longer follow-up. We found no evidence that the health guidance intervention was associated with improvement in cardiovascular risk factors.

The observed effect size of a weight reduction of approximately 0.4% (a reduction of 0.29 kg from the baseline mean weight of 71.4 kg) was modest at best. However, it was the intention-to-treat effect (an estimated effect of assignment to the health guidance intervention), and we also found that ToT effect (effect of receipt of the guidance) was 5 to 6 times greater. The observed weight loss (ToT effect) of −2.2% (1.56 kg reduction) in our study was smaller than other lifestyle interventions for obesity, such as the 6.0% weight loss seen with the Diabetes Prevention Program.^24^ This is probably because the threshold of waist circumference was relatively low; therefore, the population that received the intervention was relatively healthy. It may also be the case that the influence of an intervention for obesity implemented in the real world (effectiveness) may be smaller than what we find in randomized clinical trials (RCTs) (efficacy) because participants recruited in randomized clinical trials are usually self-selected, highly motivated individuals.

We found no evidence that health guidance intervention was associated with improvements in blood pressure, HbA_1c_ level, and LDL cholesterol level. There are several potential explanations. First, the marginal population with waist circumferences around the threshold value was relatively healthy; therefore, the magnitude of the improvement, even if it existed, might be too small to be detected even with the large sample size of our study. Second, the health guidance intervention focused on improving obesity, and improving cardiovascular risk factors was secondary. Third, given that participants were relatively healthy, the proportion of participants who required medical interventions, which may be needed to improve cardiovascular risk factors, was small. Lastly, although the health guidance intervention in Japan was implemented as a mandatory program, it has not been effectively enforced (only 15.9% of eligible participants actually received the intervention in 2017), which may explain why we did not observe clinically meaningful improvements in health outcomes.

Our findings were consistent with existing evidence^8^ that found very small, short-term (no clinically meaningful) effects of lifestyle interventions on weight loss (findings from previous studies are summarized in eAppendix M in the Supplement). Given that the exact design of lifestyle interventions varies from one to another, it is possible that more intensive programs—such as the one implemented in Japan—may be more effective than other programs. Our findings differed from a study by Nakao et al^25^ that compared individuals who attended the health guidance intervention (compliers) vs those who did not (noncompliers) and reported dramatic improvements in both weight and cardiovascular risk factors. However, compliers and noncompliers differed in ways that could not be accounted for by adjusting for only observed variables (compliers might be more motivated to improve lifestyle than noncompliers); therefore, their findings might overestimate the impact of the guidance. To address this issue, as secondary analyses, they also used the facility-level proportion of participants who underwent the health guidance intervention as an instrument in the instrumental variable method. However, facilities that attracted more health-conscious participants are likely to experience a larger improvement in health outcomes, and such violation of the exclusion restriction of the instrumental variable method leads to biased estimates. Our choice of the RD method, which is often used in situations that do not permit randomized clinical trials, leverages the fact that individuals just above and below the threshold value of the assignment variable are likely similar and that treatment assignment above the arbitrary cutoff simulates randomization. This design is another, potentially more robust, method to evaluate the association of the health guidance intervention with health outcomes.

### Limitations

Our study has limitations. First, the lack of data on more detailed information about the health guidance intervention each participant received (eg, whether participants underwent individual vs group interviews) precluded us from evaluating whether the association of the health guidance intervention varied by how it was delivered. Second, we could not identify the exact reason as to why the observed association attenuated over time. We could not disentangle 2 potential mechanisms: the guidance had only a short-term impact, as is the case with many lifestyle interventions, or it was due to treatment contamination of the study population (ie, more individuals who were just below the threshold at the initial screening gain weight and became eligible for the guidance over time). Finally, given that our study focused on corporate employees in Japan, the findings may not be generalizable to individuals who are unemployed or to populations of other countries.

## Conclusions

In summary, among working-age men in Japan, we found that the government-led national health guidance intervention was not associated with clinically meaningful or sustained weight loss. We found no evidence that health guidance intervention was associated with improvements in cardiovascular risk factors. Given the high cost of national program implementation, the intervention deployed in this intensive risk reduction program needs to be reevaluated and retooled to more effectively improve population health outcomes.

## Notes

Supplement. **eAppendix A:** The Japanese government’s guideline for the provision of the national health guidance intervention. **eAppendix B:** Assignment to the health guidance intervention based on the waist circumference value. **eFigure 1: **Flow of the screening program for obesity and cardiovascular risk factors in Japan. **eFigure 2: **Participant selection in regression discontinuity design. **eFigure 3: **Proportion of participants who were assigned to the health guidance intervention based on their value of the waist circumference. **eAppendix C:** Fuzzy RD design. **eAppendix D:** Test for manipulation of the waist circumference value. **eFigure 4: **Distribution of participants by waist circumference. **eAppendix E:** Continuity of observed covariates at the threshold level. **eTable 1: **Continuity test of observed covariates. **eAppendix F:** Robustness for bandwidth selection in the RDD analysis. **eTable 2: **Robustness for bandwidth selection in the RDD analysis. **eAppendix G:** Effect of covariates adjustment in the RD analysis. **eTable 3: **Unadjusted model. **eAppendix H:** Effect of the prior assignment in the RD analysis. **eTable 4: **Impact of guidance assignment on health outcomes in the subset without prior assignment. **eAppendix I:** Robustness for the loss to follow-up in the RD analysis. **eTable 5: **Impact of assignment to the health guidance intervention on health outcomes, using weighted RDD to account for the missing data. **eAppendix J:** Falsification test using pre-intervention outcomes. **eTable 6: **Falsification test examining the impact of the health guidance intervention on health outcomes measured one year before the screening. **eAppendix K:** Changes in drug use and lifestyle. **eTable 7: **Effect of the guidance assignment on changes in drug use, smoking, and exercise habits. **eAppendix L:** Impact of the health guidance intervention among women employees. **eTable 8: **Impact of assignment to the health guidance intervention on health outcomes among women using fuzzy regression discontinuity design. **eAppendix M:** Review of health screenings and lifestyle interventions around the world. **eTable 9: **Review of the effects of screenings and lifestyle interventions on health outcomes.
eReferences.
