# Supplementary material for: Association of the National Health Guidance Intervention for Obesity and Cardiovascular Risks With Health Outcomes Among Japanese Men
Source: JAMA Intern Med. 2020 Oct 5;180(12):1630–7. doi: 10.1001/jamainternmed.2020.4334 (PMC7536624; doi:10.1001/jamainternmed.2020.4334)
Supplement: Supplement. — eAppendix A: The Japanese government’s guideline for the provision of the national health guidance intervention. eAppendix B: Assignment to the health guidance intervention based on the waist circumference value. eFigure 1: Flow of the screening program for obesity and cardiovascular risk factors in Japan. eFigure 2: Participant selection in regression discontinuity design. eFigure 3: Proportion of participants who were assigned to the health guidance intervention based on their value of the waist circumference. eAppendix C: Fuzzy RD design. eAppendix D: Test for manipulation of the waist circumference value. eFigure 4: Distribution of participants by waist circumference. eAppendix E: Continuity of observed covariates at the threshold level. eTable 1: Continuity test of observed covariates. eAppendix F: Robustness for bandwidth selection in the RDD analysis. eTable 2: Robustness for bandwidth selection in the RDD analysis. eAppendix G: Effect of covariates adjustment in the RD analysis. eTable 3: Unadjusted model. eAppendix H: Effect of the prior assignment in the RD analysis. eTable 4: Impact of guidance assignment on health outcomes in the subset without prior assignment. eAppendix I: Robustness for the loss to follow-up in the RD analysis. eTable 5: Impact of assignment to the health guidance intervention on health outcomes, using weighted RDD to account for the missing data. eAppendix J: Falsification test using pre-intervention outcomes. eTable 6: Falsification test examining the impact of the health guidance intervention on health outcomes measured one year before the screening. eAppendix K: Changes in drug use and lifestyle. eTable 7: Effect of the guidance assignment on changes in drug use, smoking, and exercise habits. eAppendix L: Impact of the health guidance intervention among women employees. eTable 8: Impact of assignment to the health guidance intervention on health outcomes among women using fuzzy regression discontinuity design. eAppendix M: Review [file jamainternmed-e204334-s001.pdf]

Supplemental Online Content

Fukuma S, Iizuka T, Ikenoue T, Tsugawa Y. Association of the national health guidance intervention for obesity and cardiovascular risks with health outcomes among Japanese men. *JAMA Intern Med*. Published online October 5, 2020. doi:10.1001/jamainternmed.2020.4334

eAppendix A: The Japanese government's guideline for the provision of the national health guidance intervention.
eAppendix B: Assignment to the health guidance intervention based on the waist circumference value.
eFigure 1: Flow of the screening program for obesity and cardiovascular risk factors in Japan.
eFigure 2: Participant selection in regression discontinuity design.
eFigure 3: Proportion of participants who were assigned to the health guidance intervention based on their value of the waist circumference.
eAppendix C: Fuzzy RD design.
eAppendix D: Test for manipulation of the waist circumference value.
eFigure 4: Distribution of participants by waist circumference.
eAppendix E: Continuity of observed covariates at the threshold level.
eTable 1: Continuity test of observed covariates.
eAppendix F: Robustness for bandwidth selection in the RDD analysis.
eTable 2: Robustness for bandwidth selection in the RDD analysis.
eAppendix G: Effect of covariates adjustment in the RD analysis.
eTable 3: Unadjusted model.
eAppendix H: Effect of the prior assignment in the RD analysis.
eTable 4: Impact of guidance assignment on health outcomes in the subset without prior assignment.
eAppendix I: Robustness for the loss to follow-up in the RD analysis.
eTable 5: Impact of assignment to the health guidance intervention on health outcomes, using weighted RDD to account for the missing data.
eAppendix J: Falsification test using pre-intervention outcomes.
eTable 6: Falsification test examining the impact of the health guidance intervention on health outcomes measured one year before the screening.
eAppendix K: Changes in drug use and lifestyle.
eTable 7: Effect of the guidance assignment on changes in drug use, smoking, and exercise habits.
eAppendix L: Impact of the health guidance intervention among women employees.
eTable 8: Impact of assignment to the health guidance intervention on health outcomes among women using fuzzy regression discontinuity design.
eAppendix M: Review of health screenings and lifestyle interventions around the world.
eTable 9: Review of the effects of screenings and lifestyle interventions on health outcomes.
eReferences.

This supplemental material has been provided by the authors to give readers additional information about their work.

© 2020 Fukuma S et al. *JAMA Internal Medicine.*

eAppendix A: The Japanese government's guideline for the provision of the national health guidance intervention

Following is the English translation of "The implementation guideline for the national screening program and health guidance for raising awareness of metabolic syndrome" (Version 3.1) provided by the Ministry of Health, Labour and Welfare in Japan, March 2020 (https://www.mhlw.go.jp/stf/seisakunitsuite/bunya/0000161103.html).

All participants who underwent the national screening program are categorized into three risk groups (low, medium, or high) based on their estimated risk of developing cardiovascular disease (**Figure 1**). Those participants identified as low-risk for cardiovascular diseases receive the **summary report**. Those participants with a medium and high estimated risk of cardiovascular diseases are required by law to receive health guidance intervention from a trained instructor. Medium-risk participants are required to receive **moderate support**, and high-risk participants are required to receive **intensive support**.

1. Summary report (*Joho Teikyo*)

Payers (or "instructors" hired by payers or by outsourced companies to provide health guidance intervention to participants) are required to provide written feedback on the results of the health screenings (Summary report) to help increase participants' awareness of their health conditions and consider reviewing their lifestyle choices. In addition, this process aims to facilitate participants' understanding of the importance of making appointments, seeing their healthcare providers, and taking appropriate medications if necessary. This process also aims to communicate the importance of undergoing the health screening program annually.

Instead of providing uniform information to all participants, payers (instructors) are required to tailor the information for each participant based on their screening and questionnaire results. This individualized approach is likely to be more effective in raising participants' awareness and emphasizing the importance of lifestyle improvements. It is important to report the results of health screenings and questionnaires in a way that is useful for each participant so they can maintain and improve their health status, understand the importance of receiving the health screening program annually, comprehend the relationship between lifestyle changes and test results, learn the importance of lifestyle improvements, and—for those who need to see healthcare providers and receive medical treatment—understand the importance of those medical interventions.

For low-risk participants who are not required to receive health guidance intervention (those who are only eligible for Summary report), this process is a particularly valuable opportunity to increase awareness and motivate lifestyle changes to maintain health. It is also important to note that the risk of diseases, such as stroke, could be elevated even for non-obese individuals if they have other risk factors. Therefore—particularly for the following participants—it is desirable that instructors provide support to help them make lifestyle improvements, recommendations to consult healthcare providers if needed, and communicate the value of controlling lifestyle-related illnesses:

© 2020 Fukuma S et al. *JAMA Internal Medicine.*

- Those participants whose waist circumference and examination data are currently within the normal range but who are high-risk based on smoking status, dietary patterns, physical activity, etc., and who are expected to experience deterioration of their health in the future.
- Those participants whose blood pressure and laboratory test results indicate a high-risk status but are ineligible for health guidance intervention because their waist circumference (and BMI for men) do not meet the criteria for health guidance.
- Those participants who have not seen healthcare providers despite receiving recommendations to do so.
- Those participants whose health conditions are poorly controlled even though they are on appropriate medication(s).

The following types and measures of support will be selected and used based on the characteristics of the target population and the payer. For those payers who outsource the process to other companies, it is required that they include this information in all contracts because outsourcing companies must provide appropriate feedback in addition to implementing health screening programs.

- When reporting the results of the screening program, do so individually, and provide informational materials (e.g., brochures).
- If information and communication technology (ICT) is used on a daily basis in the workplace, the "Summary report" screen can be used [instead of in-person meetings].
- Informational materials (e.g., brochures) should be distributed when payers/instructors hold briefing sessions.

2. Moderate Support (*Doki-zuke Shien*)

2-1. Contents and types of support

In principle, only one interview needs to be provided for each individual. The time between the initial interview and the re-evaluation of the participant's lifestyle is typically three months. However, payers may, at their discretion, add an extra evaluation after six months or follow-up after three months of re-evaluation, depending on the condition and motivation of the participant.

Support should be provided in ways that facilitate the participant's recognition of lifestyle factors that require improvement, understanding of healthy lifestyles that should be further encouraged, and realization of the importance of taking concrete actions towards achieving better health. Based on the results of screening and survey responses on smoking status, physical activity, dietary patterns, and other lifestyle factors, support through interviews and lifestyle evaluation (evaluation conducted three months after the date of the development of the initial action plan) must be provided.

2-2. Specifics of the support provided during the interview

Individual support of 20 minutes or more per participant (30 minutes or more for remote interviews using ICT) or group support of 80 minutes or more per group (generally no more than 8 participants per group) must be provided. However, if the initial interview is conducted in multiple sessions (i.e., split into shorter sessions), the duration of the second interview can be less than 20 minutes for individual support and less than 80

© 2020 Fukuma S et al. *JAMA Internal Medicine.*

minutes for group support, depending on the results of the participants' medical examination and how the initial interview proceeded. The specific content that must be included is as follows:

- Explain the participant's need for lifestyle improvements by facilitating learning about the relationship between lifestyle factors and the test results, about metabolic syndrome and lifestyle diseases, and about recognizing the effect of lifestyle on health and the quality of life.
- Explain the benefits of improving lifestyles.
- Provide practical guidance for improving lifestyle factors, such as diet and exercise.
- Support each participant in setting behavioral goals and performance evaluation timelines, introduce them to social resources necessary for improved lifestyles, and encourage them to effectively utilize those resources.
- Teach methods of accurately measuring body weight and waist circumference.
- Guide each participant in developing action goals and action plans.

2-3. Performance evaluation

Interviews held in-person or by other modes of communication (telephone, e-mail, fax, letter, etc.) may be used for performance evaluation. If the interview is not in-person, communication must be interactive and two-way (rather than one-way from the instructor to the participants) to effectively collect useful information necessary to understand the participants' performance (e.g., enabling and hindering factors). The specific content that must be included is as follows:

- The performance evaluation should assess the effectiveness of the health guidance intervention for each participant.
- Evaluate whether behavioral goals have been achieved, and whether changes in physical conditions and lifestyle have occurred.
- If necessary, the timeline for achieving goals should be set. Also, in addition to the participant's self-evaluation of their performance, a physician, public health nurse, or nutritionist needs to evaluate the participant's performance three or more months after the development of the initial action plan.
- The results of the performance evaluation should be provided through in-person interviews or other types of communications (telephone, e-mail, fax, letter, etc.).

3. Intensive Support (*Sekkyoku-teki Shien*)

3-1. Content and types of support

Support is provided through an initial interview, followed by continuous support for three months or more. The time between the initial interview and the performance evaluation is typically three months. However, payers may, at their discretion, additionally evaluate after six months or conduct subsequent follow-up sessions three months after the initial performance evaluation, depending on the condition and motivation of the participants.

Based on the results of the screening and survey responses on smoking status, physical activity, dietary patterns, and other lifestyle factors, evaluate the progress of the support and action plan through interviews (mid-term evaluation) and performance evaluation (evaluation after three months from the date of development of the initial action plan). The specific content that needs to be included is as follows:

© 2020 Fukuma S et al. *JAMA Internal Medicine.*

- The content of the program should be such that each participant recognizes their health condition(s) and the factors they need to improve; participants should be able to continue making active efforts to improve their lifestyles.
- Based on the results of the screening and survey responses on dietary patterns, physical activity, smoking status, and other lifestyle factors, it is necessary that participants subject to intensive support make lifestyle and behavior changes (hereafter referred to as "behavioral changes"). To help participants understand the importance of improving their health, it is invaluable to recognize the environment in which participants live, and encourage them to understand the changes in their health condition(s) using the results of screening programs over time.
- Understanding and accepting the values and preferences of participants regarding their current and future health status is important. After better understanding their perspectives, encourage participants to learn the necessity of behavioral change, and support them in selecting specific action goals that can be pragmatically implemented.
- Participants, working closely with their instructor, should feel positively supported to make their own choices and to prioritize feasible action goals.
- A physician, public health nurse, or management dietitian must prepare health guidance and support plans necessary for participants to achieve their health-related goals, understand and evaluate participants' behavioral changes, and modify the health guidance intervention and support plans based on evaluation results, if necessary.
- The instructor is required to regularly support participants so they can make progress toward achieving their targeted goals.
- At the end of intensive support sessions, it is necessary to improve the participants' awareness of the importance of lifestyle factors in maintaining health status. This will help participants maintain their improved behavioral changes after the completion of the intervention.

3-2. Specifics of the support provided during the initial interview

Individual support of 20 minutes or more per participant (30 minutes or more for remote interviews using ICT), or group support of 80 minutes or more per group (generally no more than 8 participants per group) must be provided. However, if the initial interview is conducted in multiple sessions (i.e., split into shorter sessions), the duration of the second interview can be shorter, depending on the results of the screening program and how the first interview proceeded.

3-3. Specifics of continuous support for three months or more

The quantity of support is calculated using "points" (see the Table below for details about how points are assigned to each service). Payers must provide 180 points or more of support services included in Support A, or 180 points or more of services combining items included in Support A (at least 160 points) and those included in Support B. However, those who have received intensive support for two consecutive years, and those whose condition has improved in the second year compared to the first year, are deemed to have received a sufficient quantity of health guidance intervention, even if the points total less than the cut-off value.

© 2020 Fukuma S et al. *JAMA Internal Medicine.*

If more than one service (included in intensive support) is provided on the same day, only one of the services is eligible to earn points. If the same service is provided more than once on the same day, only one service is eligible to earn points. Information that is not directly related to health guidance (e.g., small talk, checking-in) may not be counted towards points.

When providing intensive support via telephone or e-mail, exchanges to request the development and submission of an action plan are not eligible to earn points.

© 2020 Fukuma S et al. *JAMA Internal Medicine.*

Structure of continuous support for three months or more

**Support A**		
Content	・Provide support based on participants' current lifestyle and progress on action plans.<br>・Provide practical guidance on lifestyle factors, such as diet and physical activity.<br>・Evaluate and revise progress on action plans in a timely manner based on evaluation results.<br>・Request that participants submit detailed descriptions of their achievements among the items included in their action plans; provide feedback based on that information.	
Type of support	Individual-level face-to-face interview	20 points for every 5 minutes (the minimum duration of the interview is 10 minutes)<br>Upper limit = 120 points
	Group-level face-to-face interview	10 points for 10 minutes (the minimum duration of the interview is 40 minutes)<br>Upper limit = 120 points
	Telephone	15 points for 5 minutes (the minimum duration of the interview is 5 minutes)<br>Upper limit = 60 points
	e-mail	40 points for one round of e-mail exchanges
**Support B**		
Content	Review the implementation of the action plan developed during the initial interview and encourage and incentivize participants to maintain the behavioral change(s) set in the action plan.	
Type of support	Individual-level face-to-face interview	10 points for 5 minutes (at least 5 minutes)<br>Upper limit = 20 points
	Telephone	5 points for 10 minutes (at least 5 minutes)<br>Upper limit = 20 points
	e-mail	5 points for one round of e-mail exchanges

3-4. Performance evaluation

In-person interviews or other types of communication (telephone, e-mail, fax, letter, etc.) may be used. When another type of communication is used, it must be interactive and two-way instead of one-way communication from the instructor to the participant. This process also may be implemented as part of the final round of continuous support. The specific contents to be included are the same as those for "moderate support."

© 2020 Fukuma S et al. *JAMA Internal Medicine.*

eAppendix B: Assignment to the health guidance intervention based on the waist circumference value

During the screening program, all participants above the threshold level of waist circumference were notified as having “central obesity.” Further, participants who meet the following criteria (A-C) are required to undergo health guidance intervention;

A. Waist circumference above the threshold level (85 cm) or body mass index above 25 kg/m^2.

B. At least one cardiovascular risk factor (hypertension, diabetes, and dyslipidemia).

C. Not receiving medications for hypertension, diabetes, and dyslipidemia.

eFigure 1: Flow of the screening program for obesity and cardiovascular risk factors in Japan

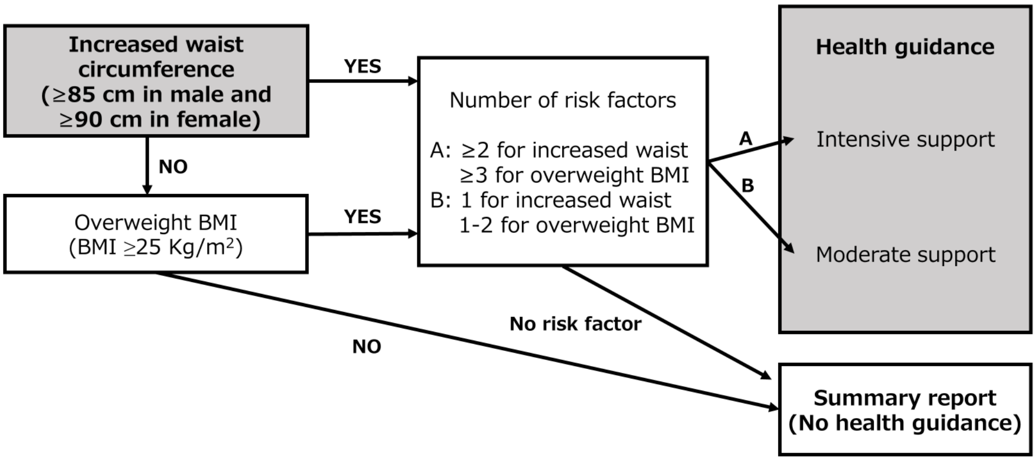

© 2020 Fukuma S et al. *JAMA Internal Medicine.*

eFigure 2: Participant selection in regression discontinuity design.

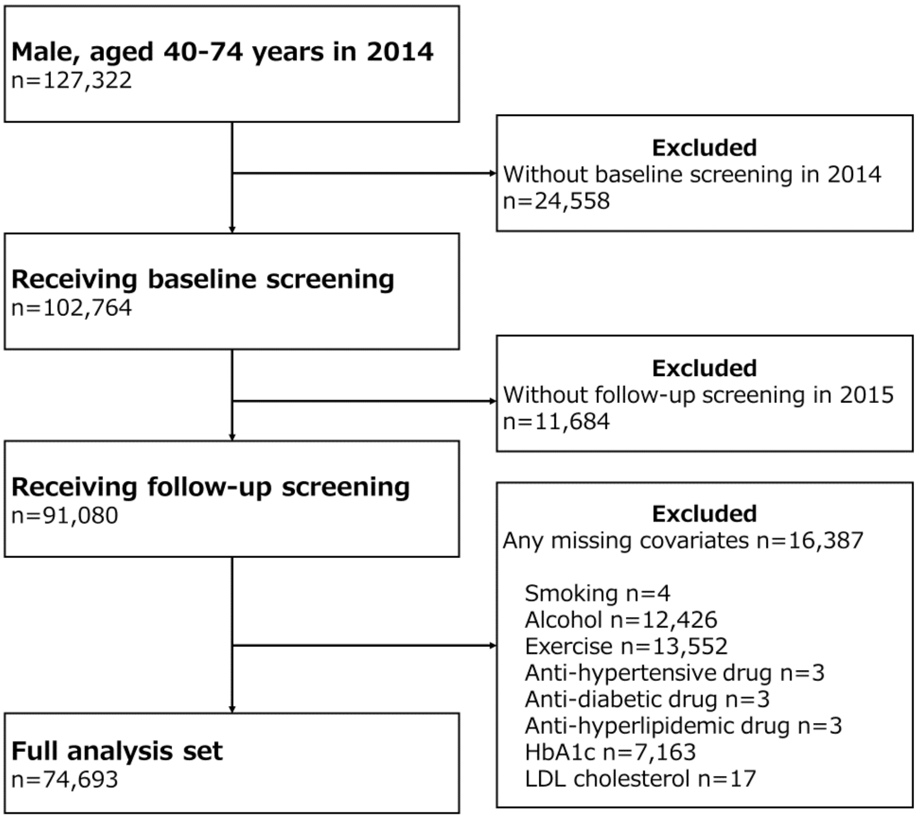

eFigure 3: Proportion of participants who were assigned to the health guidance intervention based on their value of the waist circumference

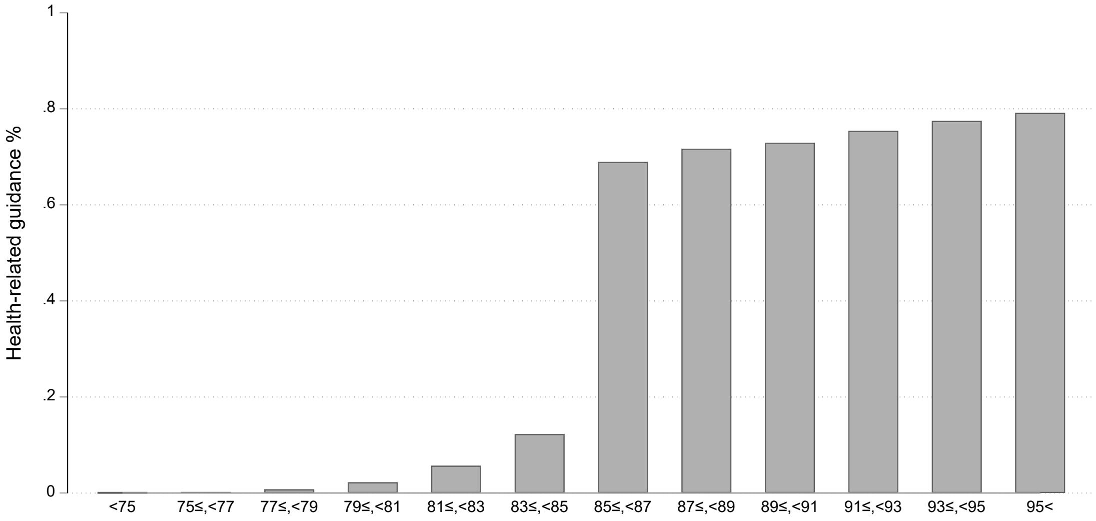

© 2020 Fukuma S et al. *JAMA Internal Medicine.*

eAppendix C: Fuzzy RD design

Fuzzy RD design consists of two stages. The first stage model is the regression analysis of the impact of the assignment variable (waist circumference value) on the probability of getting assigned to health guidance intervention. The second stage model is the regression analysis of the impact of the assignment to health guidance intervention on health outcomes. By combining these two stages, we can estimate the causal impact of the assignment to the health guidance intervention on health outcomes.

© 2020 Fukuma S et al. *JAMA Internal Medicine.*

eAppendix D: Test for manipulation of the waist circumference value

We conducted a formal test for the existence of manipulation of the assignment variable (i.e., waist circumference) using the McCrary test. We used the "rddensity" program in Stata with the local polynomial density estimators. We failed to reject the null hypothesis of a smooth density of waist circumference at the threshold (p=0.31), indicating that there is no evidence that waist circumference was manipulated by participants during the screening program.

eFigure 4: Distribution of participants by waist circumference. The vertical dashed line represents the threshold value of waist circumference (85 cm).

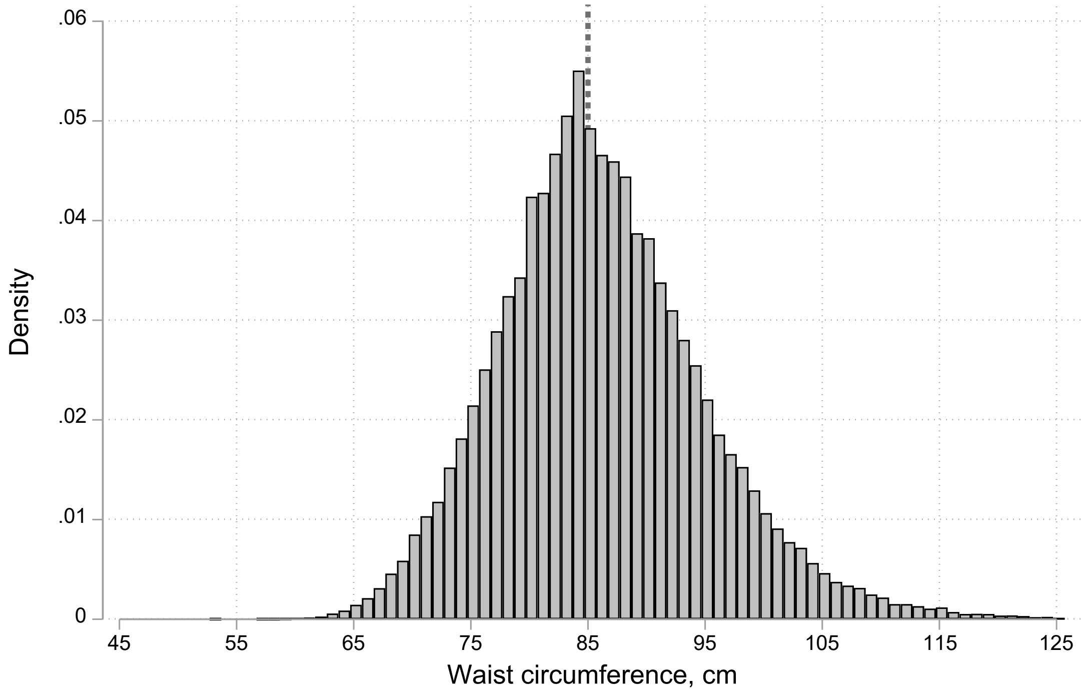

© 2020 Fukuma S et al. *JAMA Internal Medicine.*

eAppendix E: Continuity of observed covariates at the threshold level

To test the smooth continuity of observed covariates at the threshold level of waist circumference, we conducted RDD using covariates as the outcome variables and waist circumference as the explanatory variable. We did not find any discontinuity in observed covariates at the threshold of waist circumference (eTable 1).

eTable 1: Continuity test of observed covariates.

Variables	**Difference**
Age, year	+0.01 (-0.35 to +0.34, p=0.98)
Smoke, %	+1.28 (-0.82 to +2.65, p=0.22)
Moderate alcohol, %	+0.25 (-1.41 to +2.01, p=0.73)
Exercise habits, %	-0.30 (-2.53 to +2.21, p=0.90)
Anti-hypertensive drugs, %	+0.75 (-1.22 to +2.08, p=0.61)
Anti-diabetic drugs, %	+0.07 (-0.80 to +0.99, p=0.84)
Anti-hyperlipidemic drugs, %	-0.37 (-1.63 to +1.07, p=0.69)

Adjusted for age, lifestyle variables (current smoking, alcohol habits, exercise habits), systolic blood pressure, diastolic blood pressure, hemoglobin A1c, LDL cholesterol, and drug use (anti-hypertensive drugs, anti-diabetic drugs, anti-hyperlipidemic drugs) except for their own variables.

© 2020 Fukuma S et al. *JAMA Internal Medicine.*

eAppendix F: Robustness for bandwidth selection in the RDD analysis

To assess the robustness for bandwidth selection in the RDD, we used different bandwidths (60%, 80%, 120%, and 140% of optimal bandwidth). Our findings were qualitatively unaffected by the use of different bandwidths (eTable 2).

eTable 2: Robustness for bandwidth selection in the RDD analysis.

	Bandwidth	Number of participants within the bandwidth	Difference in health outcomes
Change in weight, kg	60%: 3.6 cm	26,380	-0.24 (-0.47 to +0.07, p=0.14)
	80%: 4.8 cm	32,761	-0.29 (-0.52 to -0.05, p=0.02)
	120%: 7.2 cm	46,372	-0.30 (-0.49 to -0.11, p=0.002)
	140%: 8.4 cm	50,947	-0.29 (-0.48 to -0.12, p=0.001)
Change in BMI, kg/m^2	60%: 3.6 cm	26,380	-0.13 (-0.27 to +0.01, p=0.07)
	80%: 4.8 cm	32,761	-0.14 (-0.26 to -0.02, p=0.02)
	120%: 7.2 cm	46,372	-0.13 (-0.23 to -0.04, p=0.005)
	140%: 8.4 cm	50,947	-0.12 (-0.22 to -0.05, p=0.002)
Change in waist circumference, cm	60%: 3.6 cm	26,380	-0.25 (-0.54 to +0.16, p=0.28)
	80%: 4.8 cm	32,761	-0.29 (-0.57 to +0.04, p=0.09)
	120%: 7.2 cm	46,372	-0.35 (-0.57 to -0.07, p=0.01)
	140%: 8.4 cm	50,947	-0.35 (-0.55 to -0.09, p=0.007)

Adjusted for age, lifestyle variables (current smoking, alcohol habits, exercise habits), systolic blood pressure, diastolic blood pressure, hemoglobin A1c, LDL cholesterol, and drug use (anti-hypertensive drugs, anti-diabetic drugs, anti-hyperlipidemic drugs).

© 2020 Fukuma S et al. *JAMA Internal Medicine.*

eAppendix G: Effect of covariates adjustment in the RD analysis

We assessed the effect of covariates adjustment in the RDD, comparing results with and without covariates in the regression model. Our findings were remained largely unchanged by covariates adjustment in the RDD analysis (eTable 3).

eTable 3: Unadjusted model.

	**Unadjusted**
Main outcomes	
Change in weight, kg	-0.28 (-0.48 to -0.09, p=0.004)
Change in BMI, kg/m^2	-0.12 (-0.22 to -0.03, p=0.007)
Change in waist, cm	-0.23 (-0.45 to +0.06, p=0.13)
Secondary outcomes	
Change in systolic blood pressure, mmHg	+0.72 (-0.09 to +1.97, p=0.07)
Change in diastolic blood pressure, mmHg	-0.18 (-0.96 to +0.45, p=0.48)
Change in HbA1c, %	-0.01 (-0.05 to +0.02, p=0.38)
Change in LDL cholesterol, mg/dL	+0.94 (-0.76 to +2.98, p=0.25)

We used the bandwidth of RDD of 6cm from the threshold of waist circumference.

© 2020 Fukuma S et al. *JAMA Internal Medicine.*

eAppendix H: Effect of the prior assignment in the RD analysis.

To investigate the effect of some participants having received the same guidance in the prior year, we reanalyzed the data restricting our sample to participants who were not assigned to the guidance in 2013. We found similar results to the original analysis.

eTable 4: Impact of guidance assignment on health outcomes in the subset without prior assignment.

	Main outcomes
	One year after (FY 2015)
Number of participants within bandwidth	21,290
Change in weight	
Body weight, kg	-0.36 (-0.60 to -0.04, p=0.03)
BMI, kg/m^2	-0.17 (-0.31 to -0.01, p=0.04)
Waist circumference, cm	-0.52 (-0.75 to -0.04, p=0.03)
Change in cardiovascular risk factors	
Systolic blood pressure, mmHg	+0.17 (-0.90 to +1.85, p=0.50)
Diastolic blood pressure, mmHg	-0.66 (-1.69 to +0.19, p=0.12)
Hemoglobin A1c, %	-0.03 (-0.07 to +0.01, p=0.17)
LDL cholesterol, mg/dL	+0.40 (-1.95 to +2.96, p=0.69)

We used bandwidth of RDD as 6cm from the threshold of waist circumference.
Adjusted for age, lifestyle variables (current smoking, alcohol habits, exercise habits), systolic blood pressure, diastolic blood pressure, hemoglobin A1c, LDL cholesterol, and drug use (anti-hypertensive drugs, anti-diabetic drugs, anti-hyperlipidemic drugs).

© 2020 Fukuma S et al. *JAMA Internal Medicine.*

eAppendix I: Robustness for the loss to follow-up in the RD analysis.

To assess the possibility that missing due to drop out may affect the results, we conducted a weighted RD analysis using the inverse probability of follow-up data being observed. The estimates from the weighted RD were similar to the original analysis.

eTable 5: Impact of assignment to the health guidance intervention on health outcomes, using weighted RDD to account for the missing data.

	Main outcomes	Secondary outcomes		
	One year after (FY 2015)	Two years after (FY 2016)	Three years after (FY 2017)	Four years after (FY 2018)
**Number of participants within bandwidth**	39,161	34,293	31,400	28,975
Change in weight				
Body weight, kg	-0.31 (-0.57 to -0.03, p=0.03)	-0.35 (-0.74 to +0.05, p=0.09)	-0.37 (-0.79 to +0.11, p=0.14)	-0.13 (-0.60 to +0.45, p=0.78)
BMI, kg/m2	-0.13 (-0.23 to -0.03, p=0.01)	-0.12 (-0.25 to+ 0.02, p=0.09)	-0.12 (-0.26 to +0.04, p=0.15)	-0.03 (-0.18 to +0.17, p=0.92)
Waist circumference, cm	-0.38 (-0.71 to -0.04, p=0.03)	-0.27 (-0.69 to +0.22, p=0.31)	-0.46 (-0.98 to +0.05, p=0.08)	-0.36 (-0.92 to +0.25, p=0.26)
Change in cardiovascular risk factors				
Systolic blood pressure, mmHg	-0.09 (-1.04 to 1.13, p=0.93)	-0.07 (-1.56 to +1.45, p=0.95)	-1.37 (-3.21 to -0.04, p=0.04)	-1.69 (-3.59 to -0.06, p=0.04)
Diastolic blood pressure, mmHg	-0.74 (-1.68 to -0.06, p=0.04)	-0.07 (-1.26 to +1.00, p=0.82)	-0.74 (-2.00 to +0.31, p=0.15)	-1.28 (-2.69 to -0.03, p=0.05)
Hemoglobin A1c, %	-0.001 (-0.04 to 0.04, p=0.99)	+0.01 (-0.02 to +0.06, p=0.43)	+0.01 (-0.03 to +0.05, p=0.62)	+0.01 (-0.04 to +0.06, p=0.64)
LDL cholesterol, mg/dL	+0.30 (-2.01 to 2.52, p=0.83)	-0.96 (-3.74 to +1.61, p=0.44)	-0.27 (-3.07 to +2.62, p=0.88)	+0.43 (-2.34 to +3.99, p=0.61)

We used bandwidth of RDD as 6cm from the threshold of waist circumference.
Adjusted for age, lifestyle variables (current smoking, alcohol habits, exercise habits), systolic blood pressure, diastolic blood pressure, hemoglobin A1c, LDL cholesterol, and drug use (anti-hypertensive drugs, anti-diabetic drugs, anti-hyperlipidemic drugs).

© 2020 Fukuma S et al. *JAMA Internal Medicine.*

eAppendix J: Falsification test using pre-intervention outcomes.

As the falsification test, we assessed the impact of the health guidance intervention on health outcomes measured before the receipt of the intervention (these outcomes were not supposed to be affected by the intervention, by design), and found no evidence that the guidance affected these outcomes (eTable 6). The results of this falsification test support the validity of our findings.

eTable 6: Falsification test examining the impact of the health guidance intervention on health outcomes measured one year before the screening.

	**Difference one-year before the screening**
Change in weight	
Body weight, kg	-0.09 (-0.30 to +0.15, p=0.54)
BMI, kg/m^2	-0.03 (-0.10 to +0.05, p=0.50)
Waist circumference, cm	-0.10 (-0.35 to +0.22, p=0.66)
Change in cardiovascular risk factors	
Systolic blood pressure, mmHg	-0.50 (-1.67 to +0.33, p=0.19)
Diastolic blood pressure, mmHg	-0.04 (-0.75 to +0.67, p=0.91)
Hemoglobin A1c, %	+0.005 (-0.03 to +0.04, p=0.92)
LDL cholesterol, mg/dL	+0.86 (-0.96 to +2.76, p=0.34)

We used the bandwidth of RDD of 6cm from the threshold of waist circumference.
Adjusted for age, lifestyle variables (current smoking, alcohol habits, exercise habits), systolic blood pressure, diastolic blood pressure, hemoglobin A1c, LDL cholesterol, and drug use (anti-hypertensive drugs, anti-diabetic drugs, anti-hyperlipidemic drugs).

© 2020 Fukuma S et al. *JAMA Internal Medicine.*

eAppendix K: Changes in drug use and lifestyle.

We assessed the effect of health guidance intervention on changes in the percentage of drug use (anti-hypertensive, anti-diabetic, and anti-hyperlipidemic drugs), non-smoking, and exercise habits. We found no evidence that health guidance intervention was associated with those outcomes (eTable 7). These results may explain why we observed only a small impact on weight and the lack of improvement in cardiovascular risk factors.

eTable 7: Effect of the guidance assignment on changes in drug use, smoking, and exercise habits.

Change in intermediate outcomes	**Difference, %-points**
Anti-hypertensive drugs use	-0.04 (-1.64 to +1.55, p=0.96)
Anti-diabetic drugs use	-0.05 (-0.90 to +0.87, p=0.97)
Anti-hyperlipidemic drugs use	-0.66 (-2.65 to +0.72, p=0.26)
Non-smoking	-0.18 (-2.15 to +1.18, p=0.57)
Exercise habits	-0.13 (-3.98 to +3.84, p=0.97)

We used the bandwidth of RDD as 6cm from the threshold of waist circumference.
Adjusted for age, lifestyle variables (current smoking, alcohol habits, exercise habits), systolic blood pressure, diastolic blood pressure, hemoglobin A1c, LDL cholesterol, and drug use (anti-hypertensive drugs, anti-diabetic drugs, anti-hyperlipidemic drugs).

© 2020 Fukuma S et al. *JAMA Internal Medicine.*

eAppendix L: Impact of the health guidance intervention among women employees.

We found no evidence that the health guidance intervention was associated with improvements in obesity status or cardiovascular risk factors among women, probably due to the lack of statistical power (eTable 8). The number of women who were corporate employees (11,235) was much smaller than that of male employees (74,693), and only a small percentage (11%) of female employees met the criteria of obesity and required to receive the health guidance intervention.

eTable 8: Impact of assignment to the health guidance intervention on health outcomes among women using fuzzy regression discontinuity design.

	**Main outcomes**	**Long-term outcomes**		
	**One year after screening** (2015)	**Two years after screening** (2016)	**Three years after screening** (2017)	**Four years after screening** (2018)
**Number of participants within bandwidth**	1,633	1,432	1,334	1,224
**Change in weight**				
Body weight, kg	+0.74 (-3.18 to +4.36, p=0.76)	-0.17 (-7.33 to +5.39, p=0.76)	-0.82 (-8.61 to +6.89, p=0.83)	+3.79 (-4.14 to +14.02, p=0.29)
BMI, kg/m^2	+0.40 (-1.18 to +1.87, p=0.66)	+0.25 (-2.55 to +2.47, p=0.97)	+0.05 (-2.90 to +3.10, p=0.95)	+2.04 (-1.12 to +6.36, p=0.17)
Waist circumference, cm	+2.51 (-2.82 to +9.02, p=0.30)	+6.40 (-2.16 to +17.42, p=0.13)	+0.05 (-10.00 to +8.14, p=0.84)	+8.95 (-1.89 to +24.17, p=0.09)
**Change in cardiovascular risk factors**				
Systolic blood pressure, mmHg	-1.45 (-17.54 to +14.42, p=0.85)	-24.17 (-60.94 to +5.42, p=0.10)	-8.96 (-37.97 to +19.68, p=0.53)	+1.50 (-21.08 to +28.38, p=0.77)
Diastolic blood pressure, mmHg	-5.71 (-18.98 to +4.45, p=0.22)	-11.24 (-32.35 to +6.16, p=0.18)	-0.08 (-17.71 to +17.94, p=0.99)	-0.15 (-14.50 to +17.92, p=0.84)
Hemoglobin A1c, %	-0.09 (-0.61 to +0.41, p=0.70)	-0.74 (-1.80 to -0.11, p=0.03)	-0.65 (-1.63 to +0.16, p=0.11)	-0.55 (-1.57 to +0.23, p=0.14)
LDL cholesterol, mg/dL	+5.42 (-26.15 to +35.29, p=0.77)	+19.10 (-25.92 to +68.09, p=0.38)	+7.24 (-37.78 to +61.35, p=0.64)	+16.38 (-26.81 to +66.94, p=0.40)

We used the bandwidth of RDD of 6cm from the threshold of waist circumference.
Adjusted for age, lifestyle variables (current smoking, alcohol habits, exercise habits), systolic blood pressure, diastolic blood pressure, hemoglobin A1c, LDL cholesterol, and drug use (anti-hypertensive drugs, anti-diabetic drugs, anti-hyperlipidemic drugs).

© 2020 Fukuma S et al. *JAMA Internal Medicine.*

eAppendix M: Review of health screenings and lifestyle interventions around the world.

We summarized the screenings and related lifestyle interventions that have been examined in the Cochrane systematic reviews[6]. The systematic reviews did not find any effect of improving hard outcomes such as mortality or cardiovascular events. However, there were few studies that assessed intermediate outcomes of weight loss, which reported a small, short-term reduction in weight.[7 8] Those results are consistent with our study. Regarding lifestyle intervention, there were no other interventions where a wide range of health guidance content was standardized and implemented on a national scale, as was the case for Japan's program.

eTable 9: Review of the effects of screenings and lifestyle interventions on health outcomes.

Study	Country	Screening items	Lifestyle intervention	Outcome measures
Göteborg 1963[2]	Sweden	broad	-	mortality
Kaiser Permanente 1965[3]	US	broad	-	morbidity, mortality, disability, utilization of medical care
South-East London 1967[4]	UK	broad	GP consultation every 6 months	morbidity, utilization of medical care, absence from work, mortality
Malmö 1969[5]	Sweden	broad	quit smoking	mortality
Northumberland 1969[6]	UK	not specified	-	not specified
Stockholm 1969[7]	Sweden	broad	-	mortality
Göteborg 1970[8]	Sweden	broad	usual care	CV events, mortality
WHO 1971[9]	Europe	CV risks	Guidance	CV events
Salt Lake City 1972[10]	US	broad	-	morbidity, attitude, mortality
DanMONICA 1982[11]	Denmark	broad	advice by physician	CV events, mortality
Mankato 1982[12]	US	CV risks	health education	CV risks, health behaviors
OXCHECK 1989[13]	UK	CV risks	counseling, follow-up visits	CV risks, health behaviors
Family Heart 1990[14]	UK	CV risks	repeated follow-up, lifestyle advice	CV risks, weight
Ebeltoft 1992[15]	Denmark	broad	health discussion	CV risks, weight
Inter99 1999[16]	Denmark	CV risks	individual and group counseling	CV events, mortality
Japan 2008	Japan	CV risks	initial interview, continuous support	Weight, CV risks

GP: general practitioner, CV: cardiovascular

© 2020 Fukuma S et al. *JAMA Internal Medicine.*

eReferences.

1. Krogsboll LT, Jorgensen KJ, Gotzsche PC. General health checks in adults for reducing morbidity and mortality from disease. *Cochrane Database Syst Rev.* 2019;1:CD009009.
2. Tibblin G, Welin L, Larsson B, Ljungberg IL, Svardsudd K. The influence of repeated health examinations on mortality in a prospective cohort study, with a comment on the autopsy frequency. The study of men born in 1913. *Scand J Soc Med.* 1982;10(1):27-32.
3. Cutler JL, Ramcharan S, Feldman R, et al. Multiphasic checkup evaluation study. 1. Methods and population. *Prev Med.* 1973;2(2):197-206.
4. South-East London Screening Study Group. A controlled trial of multiphasic screening in middle-age: results of the South-East London Screening Study. 1977. *Int J Epidemiol.* 2001;30(5):935-940.
5. Lannerstad O, Sternby NH, Isacsson SO, Lindgren G, Lindell SE. Effects of a health screening on mortality and causes of death in middle-aged men. A prospective study from 1970 to 1974 of mean in Malmo, born 1914. *Scand J Soc Med.* 1977;5(3):137-140.
6. Bennett AE, Fraser IG. Impact of a screening programme in general practice: a randomized controlled trial. *Int J Epidemiol.* 1972;1(1):55-60.
7. Theobald H, Bygren LO, Carstensen J, Hauffman M, Engfeldt P. Effects of an assessment of needs for medical and social services on long-term mortality: a randomized controlled study. *Int J Epidemiol.* 1998;27(2):194-198.
8. Wilhelmsen L, Berglund G, Elmfeldt D, et al. The multifactor primary prevention trial in Goteborg, Sweden. *Eur Heart J.* 1986;7(4):279-288.

© 2020 Fukuma S et al. *JAMA Internal Medicine.*

9. European collaborative trial of multifactorial prevention of coronary heart disease: final report on the 6-year results. World Health Organisation European Collaborative Group. *Lancet.* 1986;1(8486):869-872.

10. Olsen DM, Kane RL, Proctor PH. A controlled trial of multiphasic screening. *N Engl J Med.* 1976;294(17):925-930.

11. Skaaby T, Jorgensen T, Linneberg A. Effects of invitation to participate in health surveys on the incidence of cardiovascular disease: a randomized general population study. *Int J Epidemiol.* 2017;46(2):603-611.

12. Murray DM, Luepker RV, Pirie PL, et al. Systematic risk factor screening and education: a community-wide approach to prevention of coronary heart disease. *Prev Med.* 1986;15(6):661-672.

13. Imperial Cancer Research Fund OXCHECK Study Group. Effectiveness of health checks conducted by nurses in primary care: final results of the OXCHECK study. Imperial Cancer Research Fund OXCHECK Study Group. *BMJ.* 1995;310(6987):1099-1104.

14. Family Heart Study Group. Randomised controlled trial evaluating cardiovascular screening and intervention in general practice: principal results of British family heart study. Family Heart Study Group. *BMJ.* 1994;308(6924):313-320.

15. Engberg M, Christensen B, Karlsmose B, Lous J, Lauritzen T. General health screenings to improve cardiovascular risk profiles: a randomized controlled trial in general practice with 5-year follow-up. *J Fam Pract.* 2002;51(6):546-552.

16. Jorgensen T, Jacobsen RK, Toft U, Aadahl M, Glumer C, Pisinger C. Effect of screening and lifestyle counselling on incidence of ischaemic heart disease in general population: Inter99 randomised trial. *BMJ.* 2014;348:g3617.

© 2020 Fukuma S et al. *JAMA Internal Medicine.*
